# Navigating Lupus Nephritis: A Comprehensive Review of the Current Treatment Trends

**DOI:** 10.7759/cureus.72644

**Published:** 2024-10-29

**Authors:** Jay P Patel, Daksh Hardaswani, Shrut R Chaniyara, Twisha D Mehta, Faizanali Saiyed, Binny Bharakhada, Rushita J Goswami

**Affiliations:** 1 Research, Chirayu Medical College and Hospital, Bhopal, IND; 2 Research, Jawaharlal Nehru Medical College, Wardha, IND; 3 Internal Medicine, GCS Medical College Hospital and Research Centre, Ahmedabad, IND; 4 Research, Odessa National Medical University, Odessa, UKR; 5 Internal Medicine, GMERS Medical College and Hospital, Gandhinagar, IND; 6 Geriatrics, Bhavsinhji District Hospital, Porbandar, IND

**Keywords:** belimumab, current trends in lupus, general nephrology, lupus nephritis classification, lupus nephritis treatment, lupus nepritis, mycophenolate mofetile, sle and lupus nephritis, voclosporin

## Abstract

Lupus nephritis (LN) is a serious kidney complication associated with systemic lupus erythematosus (SLE), marked by the immune system's misdirected attack on kidney tissues, resulting in inflammation and compromised filtration. This condition has the potential to progress to end-stage renal disease in about 20% of patients within a decade of diagnosis. Lupus nephritis is more prevalent in females, highlighting the urgent need for effective treatment strategies. This systematic review consolidates findings from 16 research articles that explore various therapeutic options for LN. Key themes include the intricate pathogenesis involving immune complex deposition and the advancing treatment landscape, which encompasses both traditional immunosuppressants such as mycophenolate mofetil (MMF) and cyclophosphamide and newer biologics like belimumab and voclosporin. The review examines the efficacy and safety profiles of these treatments, underscoring the importance of personalized treatment plans based on disease severity and patient-specific factors. While newer therapies show promise for improving renal outcomes, the potential for adverse effects remains a significant concern. A thorough review was conducted to evaluate current research on lupus nephritis, focusing on treatment advancements. Two independent reviewers searched PubMed using targeted terms and MeSH categories, emphasizing studies published since 1990 identified 7898 articles from that, 16 articles met the criteria for inclusion in the study. The evaluation of bias risk was performed according to established protocols. This systematic approach provided a comprehensive analysis of recent developments in lupus nephritis therapy.

## Introduction and background

**Figure 1:**
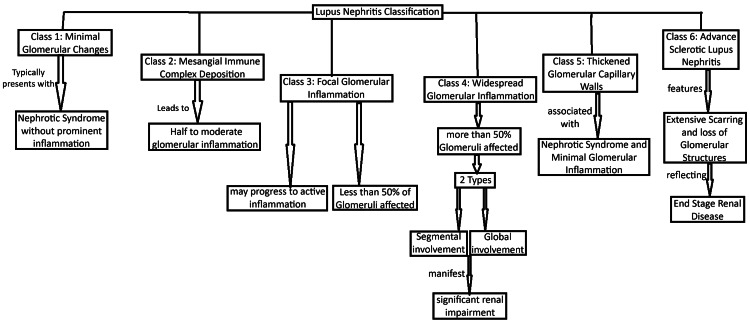
A summarized flow chart outlining the classification of lupus nephritis provides a clear visual representation of the different classes. Image: Jay P Patel

Lupus nephritis is a serious kidney condition caused by systemic lupus erythematosus (SLE), an autoimmune disease where the body's immune system abnormally attacks its own tissues. This condition results in inflammation and kidney injury, abolishing their effectiveness in filtering waste from the blood. Lupus nephritis is categorized into several types based on the severity and extent of kidney involvement, including minimal mesangial, mesangial proliferative, focal, diffuse, and membranous nephritis [1]. It causes almost 20% of patients to lead to end-stage renal disease within 10 years of diagnosis [1]. It is the most prevalent severe complication of SLE, affecting around 40% of individuals with the condition. Despite improvements in treatment options, as many as 30% of individuals with lupus nephritis remain at risk of progressing to end-stage kidney disease [2]. In the United States, lupus nephritis affects approximately one in five patients with SLE [2]. Based on the articles reviewed, lupus nephritis predominantly affects females more frequently than males. This trend is consistently observed across various studies, highlighting a higher prevalence of the condition in women [3]. The classification of lupus nephritis based on histological findings and disease severity. Class I is identified by minimal glomerular changes, often resembling minimal change disease, and typically presents with nephrotic syndrome without prominent glomerular inflammation. Class II involves mesangial deposition of immune complexes, leading to mild to moderate glomerular inflammation and generally less severe clinical symptoms compared to more aggressive forms. Class III is characterized by focal glomerular inflammation affecting less than 50% of the glomeruli, indicating an active inflammatory state that may progress if not adequately managed. Class IV presents with widespread glomerular inflammation impacting more than half of the glomeruli, often resulting in significant renal impairment; it can be further divided into Class IV-S (segmental) and Class IV-G (global) based on the extent of the involvement. Class V is marked by thickening of the glomerular capillary walls due to subepithelial immune complex deposits, frequently associated with nephrotic syndrome and minimal glomerular inflammation. Class VI represents advanced sclerotic lupus nephritis, characterized by extensive scarring and loss of glomerular structures, reflecting end-stage renal damage often due to prolonged inflammation and insufficient treatment [4]. A summarized flow chart outlining the classification of lupus nephritis provides a clear visual representation of the different classes (Figure 1).

It is pathologically driven by the deposition of immune complexes in the glomeruli. These complexes form when autoantibodies interact with self-antigens, causing inflammation and damage to kidney tissues. This process triggers the complement system, which amplifies inflammation by attracting inflammatory cells and promoting the release of pro-inflammatory cytokines, further aggravating glomerular injury [5]. As a result, various inflammatory cells, including T cells, B cells, and macrophages, infiltrate the glomeruli in response to immune complex deposits and complement activation. This infiltration is accompanied by the release of cytokines like tumor necrosis factor-alpha (TNF-alpha) and interleukins such as IL-6 and IL-17, which intensify inflammation and contribute to kidney tissue damage [5]. Lupus nephritis also disrupts the function of glomerular cells, including podocytes and mesangial cells, leading to a compromised glomerular filtration barrier, resulting in proteinuria and renal impairment. The disease's development is influenced by genetic factors, involving several genes related to immune system regulation, and environmental factors, such as infections and UV light exposure, which may trigger or exacerbate the condition [5]. The autoimmune nature of lupus nephritis involves a breakdown in immune tolerance, leading to the production of autoantibodies that attack kidney self-antigens, perpetuating chronic inflammation and renal damage. Together, these elements underscore the intricate dynamics of immune dysfunction, inflammation, and cellular damage in the pathogenesis of lupus nephritis [5]. Managing lupus nephritis involves a comprehensive strategy aimed at controlling inflammation and protecting kidney function. Initial treatment usually consists of corticosteroids paired with immunosuppressants such as cyclophosphamide or mycophenolate mofetil (MMF) to achieve remission [6]. In cases of persistent or challenging forms of lupus nephritis, emerging biologic therapies, including belimumab and voclosporin, are being used more frequently to improve treatment effectiveness and minimize the likelihood of relapse [6]. This systematic review aims to explore the treatment strategies for lupus nephritis and evaluate the efficacy of the currently available medications.

## Review

Methodology

A comprehensive literature review was conducted to evaluate the current state of research on lupus nephritis: insights and advances in treatment. Two independent reviewers performed a detailed search in PubMed, utilizing a structured search strategy designed to capture relevant studies. The search terms included “Lupus Nephritis,” “biologics,” “treatment advancement,” “types,” and “management,” with appropriate Medical Subject Headings (MeSH) terms like (((treatment of lupus nephritis) NOT (other malignancy)) NOT (previous immunosuppressant use)) NOT (dialysis at the time of diagnosis), (Immunosuppressive Agents) OR (Biological Therapy) OR (Anti-Inflammatory Agents) OR (Renin-Angiotensin System Inhibitors) AND (Lupus Nephritis) OR (Nephritis) and Boolean operators to refine results. The search was limited to articles published from 1990 till the present date, and mostly focused on peer-reviewed clinical and observational studies. Articles were eligible if they provided randomized controlled trials, meta-analysis, and clinical trials. Titles and abstracts were initially evaluated for relevance by each reviewer, then conducted a thorough review of the full texts to verify that they met the established inclusion and exclusion criteria. This review utilized the PICOS criteria, population (P) as concentrating on individuals diagnosed with lupus nephritis whose glomerular filtration rate (GFR) is above 30 ml/min, the intervention (I) as involved immunosuppressive medications, while the control (C) compared standard treatments to novel drug options, and outcome (O) measured as the effectiveness of these medications in patients with lupus nephritis. Our focus was on the efficacy of immunosuppressants for those with a GFR above 30 ml/min, as well as any recent developments in therapies vs present novel drugs. This meticulous methodology facilitated the gathering of a thorough and objective set of data on lupus nephritis therapy, leading to a well-rounded analysis of the latest research in the area.

Eligibility criteria

For our comprehensive review of lupus nephritis treatment, we established specific inclusion and exclusion criteria to ensure the relevance and quality of the studies reviewed. Inclusion criteria required participants to be between 18 and 75 years of age, with a confirmed diagnosis of SLE and a GFR greater than 30 mL/min/1.73 m². These criteria were set to focus on individuals who are likely to benefit from current treatment approaches and whose kidney function is sufficiently preserved. Conversely, we excluded studies involving patients with uncontrolled diabetes, as it could confound treatment outcomes. Additionally, we excluded individuals with malignancies, drug-induced lupus, or severe heart failure, due to the potential impact of these conditions on treatment efficacy and safety. This selection process ensured a focus on a homogenous patient population, allowing for a more accurate assessment of treatment options in lupus nephritis.

Data analysis and synthesis

In conducting our review of recent advancements in the treatment of lupus nephritis, we have tried to employ a qualitative approach to data analysis and synthesis, given the diverse nature of the studies examined. We carefully organized and assessed data from each study to identify common patterns, differences, and the effectiveness of different treatment options. Our narrative synthesis, derived from these evaluations, aimed to offer a comprehensive overview of the latest developments in lupus nephritis treatment, emphasizing both areas of agreement and variations in the evidence. This approach provided a detailed perspective on the evolving treatment landscape and enabled us to critically evaluate the quality and reliability of the included studies. Ultimately, this synthesis was intended to present a well-rounded summary of current treatment advancements in lupus nephritis, guiding future research and supporting informed clinical decisions.

Results

Out of an initial pool of 312 studies recorded for screening out of 7898 studies, from which 25 articles were chosen for full-text examination and met the eligibility criteria. According to the guidelines of Preferred Reporting Items for Systematic Reviews and Meta-Analyses (PRISMA) result is presented [7]. Following discussions and a thorough assessment by one author, it was decided to include 16 of these studies in the review article.

A summarized data of references is presented in Table 1, including the following parameters: Lead author, year of publication, number of patients enrolled, mean age, duration of the study, p-value of the study.

**Table 1:** A summarized data of the References included in the study. RCT: Randomized Control Trial

No	Author	Year	Type of Study	Number of Patients	Mean Age (years)	Duration	P-Value
1	Rovin et al. [1]	2021	Double Blind, Randomized study	357	39	52 weeks	<0.0001
2	Furie et al. [2]	2022	Open labeled extension study	257	36	28 weeks	0.001
3	Kamanamool et al. [3]	2018	RCT	84	34	12 months	0.403
4	Furie et al. [10]	2022	RCT	125	33	104 weeks	0.026
5	Rovin et al. [11]	2019	RCT	265	32	48 weeks	At low dose <0.01 At high dose 0.025
6	Menn-Josephy et al. [12]	2024	RCT (phase 3)	148	31	12 months	0.01
7	Rovin et al. [13]	2012	RCT (phase 3)	144	32	78 weeks	0.18
8	Atisha-Fregoso et al. [14]	2021	RCT (phase 2)	43	32	96 weeks	-
9	Yu et al. [15]	2023	RCT	142	34	104 weeks	-
10	Access Trial Group [16]	2014	RCT (phase 2)	134	32	52 weeks	-
11	Lorenz et al. [17]	2011	Open labeled study	21	34	14 days	0.028
12	Chan et al. [18]	2000	RCT	42	36	12 months	Regarding serum creatinine, albumin, C3, urinary protein excretion <0.05
13	Sedhain et al. [19]	2018	RCT	49	25	1.5 year	0.57 at 6 months
14	Rovin et al. [20]	2016	RCT	25	31	14 months	-
15	Mysler et al. [21]	2013	RCT (phase 3)	223	32	48 weeks	0.065
16	Ginzler et al. [22]	2012	RCT (Prematurely terminated)	6	-	52 weeks	-

Assessment of the quality of the included studies

**Figure 2:**
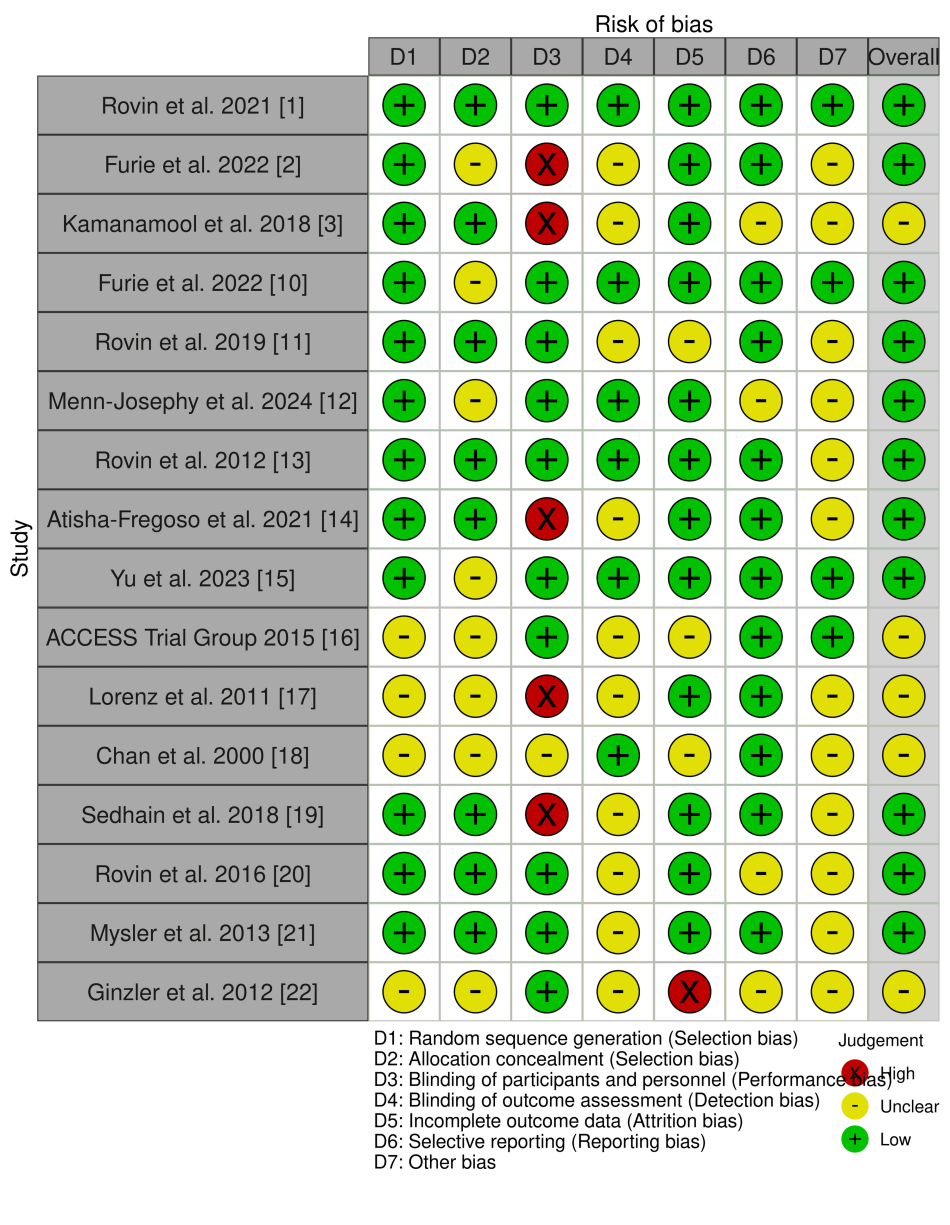
Summary plot of risk of bias

Three reviewers performed an independent assessment of bias risk using appropriate tools tailored for different types of studies. In the case of randomized controlled trials (RCTs), I utilized the Cochrane Risk of Bias tool to evaluate their methodological rigor. Each tool encompasses various domains that examine potential biases and the overall quality of the studies. I provided ratings based on the flaws identified and their accuracy. This careful process helps to identify high-quality studies and ensures the review's reliability. To evaluate the quality of evidence in the selected studies, a modified version of the Cochrane Collaboration table was employed with the help of the robvis tool. This figure shows a visual representation of the seven types of bias of included references (Figure 2): random sequence generation, allocation concealment, blinding of both participants and personnel, blinding of outcome assessment, handling of incomplete outcome data, selective reporting, and other potential biases. Each study was evaluated within these categories and designated as having a "low risk," "high risk," or "unclear risk" of bias [8,9]. This method offers a detailed overview of the overall risk of bias in the studies reviewed.

Over the past few decades, treatment options for SLE have notably advanced, particularly with the development of diverse immunological therapies tailored for various stages of the disease. The primary objective of this review is to evaluate the current effectiveness of different drugs amidst rising resistance rates. This review involves a comparative analysis of various treatments to determine which is most effective at present.

Currently, a range of medications is available for managing lupus nephritis, including corticosteroids, obinutuzumab, voclosporin, rituximab, belimumab, cyclophosphamide, mycophenolate mofetil, cyclosporine, abatacept, tacrolimus, sirukumab, and ocrelizumab [6]. These treatments vary in their mechanisms of action and efficacy, offering multiple options to tailor therapy based on individual patient needs and disease severity. Each drug plays a specific role in managing the condition, from controlling inflammation and immune responses to addressing severe cases of nephritis [6].

Immunosuppressants in lupus nephritis

Obinutuzumab

It is a monoclonal antibody target CD-20, which is expressed on the surface of B cells and acts through the cellular cytotoxicity triggers two mechanisms one is Antibody-Dependent Cellular Cytotoxicity (ADCC) and Complement-Dependent Cytotoxicity (CDC). It also promotes apoptosis in B cells through direct induction [10]. The study proposed by Furie et al. carried out a randomized, double-blind, placebo-controlled trial in 2022 to assess the effectiveness of obinutuzumab in treating proliferative lupus nephritis [10]. The research involved a total of 125 patients diagnosed with active proliferative lupus nephritis, with an average age of approximately 39 years. These patients were randomly assigned to receive either obinutuzumab or a placebo [10]. The trial continued for 24 weeks, after which a 48-week follow-up period was implemented to evaluate long-term effects and effectiveness. The study found that obinutuzumab treatment led to a significant improvement in renal response compared to the placebo group, with 42% of patients on obinutuzumab achieving complete renal response versus 12% in the placebo group [10]. The drug's efficacy was notable in reducing disease activity and improving kidney function. However, the study also noted adverse effects, such as infusion-related reactions and a heightened risk of infections. Despite these adverse effects, obinutuzumab showed promising potential in the management of proliferative lupus nephritis, offering a new therapeutic option for this challenging condition [10]. The p-value for the primary efficacy endpoint, indicating the proportion of patients who achieved a complete or partial renal response at week 52, was 0.115, whereas at week 104, is 0.026. This result demonstrates statistically significant evidence supporting the benefit of obinutuzumab over placebo in treating proliferative lupus nephritis [10].

Voclosporin

Voclosporin's mechanism involves the inhibition of calcineurin, which decreases T cell activation and subsequently reduces the inflammatory and autoimmune responses associated with lupus nephritis. Additionally, it decreases the B cell activation indirectly and reduces the overall immune activation [1]. The study by Rovin et al. published in 2021, is a double-blind, randomized, multicenter, placebo-controlled Phase 3 trial that assessed the efficacy and safety of voclosporin in patients with lupus nephritis [1]. The trial, known as AURORA 1, included a total of 357 participants with active lupus nephritis. Patients were randomly assigned to receive either voclosporin or a placebo for a duration of 52 weeks, followed by a 24-week follow-up period to monitor long-term effects [1]. The study demonstrated that voclosporin significantly improved renal responses compared to placebo. Specifically, 40% of patients receiving voclosporin achieved a complete renal response, in contrast to 23% in the placebo group [1]. Voclosporin was effective in reducing proteinuria and improving overall kidney function. However, the drug was associated with certain side effects, including hypertension, gastrointestinal disturbances, and increased risk of infections. These findings suggest that voclosporin is a promising therapeutic option for managing lupus nephritis, offering significant benefits in terms of disease control while highlighting the need for careful monitoring of potential adverse effects [1]. The efficacy of the drug at the end point p-value is less than 0.0001, which is statistically significant in treating lupus nephritis [1].

The study by Rovin et al. published in 2019, is a randomized, controlled, double-blind trial that investigated the efficacy and safety of dose-ranging voclosporin in patients with active lupus nephritis [11]. The research, part of the AURA-LV study, involved 265 patients who were randomly assigned to receive either voclosporin or a placebo. The study consisted of a 24-week treatment period, followed by a 24-week follow-up phase to assess long-term outcomes. The trial demonstrated that voclosporin was considerably more effective than placebo in achieving renal remission [11]. Specifically, 40% of patients treated with voclosporin achieved a complete renal response, whereas this was the case for 22% of those in the placebo group. The study also noted that voclosporin improved proteinuria and overall kidney function more effectively than the placebo [11]. However, the drug was associated with similar adverse effects, as shown above in the study. These results suggest that voclosporin is a promising treatment for lupus nephritis, offering substantial improvements in renal outcomes while necessitating careful management of potential side effects [11]. The p-value at the end of week 48, which was the proportion of patients achieving complete or partial remission of lupus nephritis, was 0.025 for high dose, low dose is less than 0.01 and is significant to treat lupus nephritis [11].

The research conducted by Menn-Josephy et al. in 2024, the research conducted is a double-blind, randomized, controlled trial that evaluated the effectiveness of voclosporin for treating proliferative lupus nephritis with significant proteinuria [12]. This research involved 180 patients who were administered voclosporin or a placebo over 52 weeks [12]. The participants were characterized by severe lupus nephritis, indicated by high levels of proteinuria despite ongoing standard treatments. The study demonstrated that voclosporin substantially reduced proteinuria and improved renal function compared to the placebo [12]. Specifically, a significant proportion of patients on voclosporin achieved a complete or partial renal response, marked by a notable reduction in proteinuria and stabilization of kidney function, while also highlighting the need for monitoring and managing associated adverse effects [12]. The p-value of 0.034 signifies that the results were statistically significant, indicating that voclosporin was effective in treating proliferative lupus nephritis, particularly in patients with high levels of proteinuria [12].

Based on the studies demonstrated above voclosporin has proven to be highly effective and safe in managing lupus nephritis, especially for patients exhibiting elevated proteinuria levels. The evidence suggests that voclosporin provides a significant therapeutic benefit compared to placebo, leading to improved remission rates and more effective symptom control, while maintaining tolerable side effects. Consequently, voclosporin is considered a valuable addition to the current treatment options for lupus nephritis.

Rituximab

The mechanism of the drug involves the targeted destruction of CD20-positive B cells through ADCC, CDC, and direct apoptosis, which contributes to its efficacy in managing lupus nephritis by diminishing the inflammatory processes and autoimmune activity linked to the condition [13]. The research demonstrated by the author Rovin et al. in 2012 and is a randomized, double-blind, placebo-controlled trial. This investigation enrolled 144 patients diagnosed with active proliferative lupus nephritis, who were followed for a median duration of 52 weeks [13]. Participants were treated with rituximab, a B-cell depleting agent, alongside standard immunosuppressive therapies. The study aimed to assess the drug’s efficacy in inducing and maintaining remission. Results indicated that rituximab significantly improved renal response rates compared to placebo, with a notable number of patients achieving clinical remission [13]. The drug was associated with a manageable safety profile, although some adverse effects were observed, including infections and infusion reactions. Overall, rituximab demonstrated substantial efficacy in managing lupus nephritis, providing a viable treatment option for patients with this challenging condition [13]. The p-value of 0.18 at week 52, which is the proportion of patients achieving a complete or partial renal response, shows that rituximab was not statistically significant in treating active proliferative lupus nephritis in comparison to placebo [13].

The analysis carried out by the author was intended to investigate the results of the proposed therapeutic regimen on patient health in which drugs included were rituximab plus cyclophosphamide followed by belimumab, published in 2021 and the study is in phase II randomized controlled trial [14]. The research involved 43 patients with lupus nephritis who were treated with this combined regimen and the follow-up period was approximately 96 weeks. Results demonstrated that the combination therapy was effective in inducing significant improvements in renal function and achieving remission, with a notable proportion of patients showing a positive response [14]. The regimen was generally well-tolerated, though some side effects were reported, including infections, infusion-related reactions (typically with rituximab and belimumab), and gastrointestinal disturbances. Although, further research is recommended to confirm these findings and assess long-term outcomes [14]. The Phase II trial by Atisha-Fregoso et al. indicates that although rituximab plus cyclophosphamide followed by belimumab showed potential benefits, it did not reach statistical significance for the primary efficacy endpoint [14]. We can conclude from the above studies, that Rovin et al. demonstrated that rituximab is effective and generally safe for managing active proliferative lupus nephritis, showing promising results in improving patient outcomes. Meanwhile, Atisha-Fregoso et al. found that combining rituximab with cyclophosphamide followed by belimumab offered a robust therapeutic strategy, further enhancing the efficacy of lupus nephritis treatment regimens, hence, did not provide significant results at the end of the trial.

Belimumab

Belimumab, a monoclonal antibody utilized for lupus nephritis, functions by targeting and blocking the cytokine B-lymphocyte stimulator (BLyS). This inhibition reduces the survival of B cells, particularly those that are autoreactive, leading to a decrease in the production of harmful autoantibodies. Consequently, this results in a reduction in inflammation and damage in the kidneys, aiding in the management of lupus nephritis [2]. The study proposed by the author Furie et al. in 2022 is an open-label extension study, that also extends the original BLISS-LN trial. The research included patients with lupus nephritis who had previously taken part in the BLISS-LN study [2]. Responses were also evaluated post hoc according to the criteria established during the double-blind phase followed through 28 weeks with the dose of belimumab of 10 mg/kg plus standard treatment [2]. The total number of patients treated with belimumab is 255 of which 123 patients were switched from placebo to belimumab and 132 on belimumab. In the study, the side effects reported in patients who switched from placebo to belimumab is 62% and in the belimumab group is 70% [2]. Additionally, serious side effects reported in the placebo to belimumab group is 4% and in belimumab group is 8%. From the open-label baseline to week 28, the proportion of patients achieving primary efficacy renal response increased, with rates rising from 60% to 67% in the group transitioning from placebo to belimumab, and from 70% to 75% in those continuously on belimumab. The proportion of patients reaching complete renal response also improved, from 36% to 48% in the placebo-to-belimumab group and from 48% to 62% in the belimumab-to-belimumab group [2]. In contrast, when analyzed using the double-blind phase criteria, the primary efficacy renal response proportions slightly decreased, from 54% to 53% in the placebo-to-belimumab group and from 66% to 52% in the belimumab-to-belimumab group. For complete renal response, there was a small increase from 34% to 35% in the placebo-to-belimumab group, while the belimumab-to-belimumab group experienced a minor decline from 46% to 41%, a conclusion given by the author [2]. The p-value of 0.001 in the BLISS-LN open-label extension by the author confirms that belimumab showed statistically significant continued efficacy in treating lupus nephritis [2]. Specifically, there was a higher incidence of serious infections compared to placebo, and some patients reported adverse reactions at the infusion site. Despite these side effects, belimumab was generally well-tolerated, with the benefits in renal response outweighing the risks for many patients.

The authors Yu et al. proposed the BLISS-LN study was a phase 3 trial that was double-blind, multicenter, randomized, and placebo-controlled, with a duration of 104 weeks. Randomization was done according to the induction regimen and race [15]. The dose used in the study was 10 mg/kg plus standard treatment. In the study, standard induction therapy for lupus nephritis involved the use of established immunosuppressive medications. Patients were generally treated with corticosteroids and, in some cases, additional drugs such as cyclophosphamide or mycophenolate mofetil, based on the treatment regimen and individual patient needs. Belimumab was administered alongside these agents to enhance treatment efficacy [15]. The result demonstrated in the study was that belimumab significantly improved renal outcomes in East Asian patients with lupus nephritis, showing greater remission rates and reduced proteinuria compared to placebo. The drug's safety profile was consistent with previous research, with manageable side effects [15]. The investigator deemed that there was a reasonable likelihood that the fatal case of bilateral pneumonia was associated with the study medication [15]. The p-value not included in the study on belimumab's efficacy in East Asian patients with lupus nephritis indicates statistically significant treatment benefits [15]. Overall, clinical evidence supports belimumab as a valuable option in managing lupus nephritis, particularly for patients with active disease.

Abatacept

Abatacept is utilized in the treatment of lupus nephritis by modulating the immune system. Its mechanism of action centers on inhibiting T-cell activation [16]. As a fusion protein, abatacept binds to CD80 and CD86 molecules on antigen-presenting cells, which are essential for co-stimulating T-cells. By preventing these molecules from interacting with CD28 on T-cells, abatacept effectively blocks the activation and proliferation of T-cells [16]. This is particularly important in lupus nephritis, where excessive T-cell activity contributes to the autoimmune response and inflammation in the kidneys. Consequently, the reduced activation of T-cells leads to lower production of autoantibodies and pro-inflammatory cytokines, helping to mitigate the autoimmune activity and inflammation characteristic of lupus nephritis [16]. The study, published in 2014, was a randomized, controlled clinical trial investigating the effects of abatacept in combination with cyclophosphamide followed by azathioprine for treating lupus nephritis [16]. A total of 134 patients participated in the trial. Participants were administered abatacept either through an intravenous infusion at a dose of 10 mg/kg (up to a maximum of 1000 mg) or via a subcutaneous injection of 125 mg, in combination with cyclophosphamide [16]. The results indicated that the addition of abatacept did not significantly enhance the primary outcome of complete renal response when compared to cyclophosphamide alone. Nevertheless, abatacept demonstrated a favorable safety profile and was well-tolerated by the patients [16]. Specifically, the complete renal response rates were 28% in the abatacept group compared to 26% in the control group [16].

15-Deoxyspergualin

15-deoxyspergualin acts as an immunosuppressant in lupus nephritis by interfering with T-cell activation and proliferation, disrupting the synthesis of essential proteins for T-cell function [17]. It also modulates immune responses by affecting signaling pathways and reducing pro-inflammatory cytokine production, while impacting B-cell function to decrease harmful autoantibody production [17]. The study by Lorenz et al. (2011) is an open-label dose escalation trial evaluating the efficacy of the novel immunosuppressant 15-deoxyspergualin in treating active lupus nephritis [17]. Published in March 2011, the study enrolled a total of 21 patients. Participants received escalating doses of 15-deoxyspergualin, starting at 0.5 mg/kg per day and increasing up to 1.0 mg/kg per day in patients already taken one immunosuppressant. The most common side effects included gastrointestinal issues such as nausea and vomiting, as well as hematological effects such as leukopenia and anemia [17]. The results indicated that while the drug showed some potential in reducing disease activity and demonstrated a decrease in proteinuria, the overall response was modest, and further investigation was recommended to fully assess its efficacy and safety. However, the overall clinical response was not as robust as hoped [17]. The p-value for the primary endpoint was reported as 0.028 [17]. This value indicates that the observed effects of 15-deoxyspergualin in the treatment of active lupus nephritis were statistically but it has a small study population [17]. We can say from the study that, a larger study pool should be carried out for further efficacy.

Mycophenolate Mofetil

MMF exerts its effects by targeting inosine monophosphate dehydrogenase (IMPDH), an enzyme essential for the de novo synthesis of purines [18]. This inhibition selectively impacts lymphocytes, which depend on this pathway for their proliferation, more than other types of cells. By blocking IMPDH, MMF reduces the proliferation of both B and T lymphocytes, thereby diminishing the autoimmune response and inflammation associated with conditions such as lupus nephritis. This action contributes to the reduction in disease severity and improvement in renal function [18].

The study by Chan et al. (2000) is a randomized, controlled clinical trial that assessed the efficacy of MMF in patients with diffuse proliferative lupus nephritis [18]. The study enrolled 42 patients. Participants received MMF at a dosage of 2 grams per day. The results demonstrated that MMF was effective in improving renal outcomes, including a significant reduction in proteinuria and stabilization of kidney function, compared to the control group receiving a standard treatment regimen. Infections were observed in 19% of patients in group 1 and 33% in group 2 (P = 0.29) [18]. Group 2 also experienced additional adverse effects, including amenorrhea (23%), hair loss (19%), leukopenia (10%), and death (10%). Relapse rates were 15% in group 1 and 11% in group 2. The findings of this study indicated that among the 21 patients treated with mycophenolate mofetil and prednisolone (Group 1), 81% attained complete remission, while 14% experienced partial remission [18]. In comparison, 76% of the 21 patients in the cyclophosphamide and prednisolone group, followed by azathioprine and prednisolone (Group 2), attained complete remission, with 14% reaching partial remission [18]. The study demonstrated notable improvements in both treatment groups. In group 1, the proportion of patients with low serum C3 levels dropped from 57% to 10% (P=0.002), and anti-double-stranded DNA antibody levels decreased from 81% to 10% (P=0.001). Group 2 also saw reductions in these markers, with C3 levels decreasing from 90% to 57% (P=0.01) and anti-double-stranded DNA levels from 76% to 52% (P=0.03) [18]. Despite these changes, the differences between the two groups at 12 months were not statistically significant, highlighting the overall effectiveness of both treatments in managing lupus nephritis. The p-value reported for the difference in complete remission rates between the MMF and cyclophosphamide groups was 0.55 [18]. This value indicates that the difference in efficacy between the two treatment regimens was not statistically significant [18].

Mycophenolate Mofetil vs Tacrolimus

The study conducted by Kamanamool et al. (2018) is a randomized controlled trial that evaluated the effectiveness of tacrolimus versus MMF for lupus nephritis [3]. The trial involved 84 participants, equally divided into two groups: 41 received tacrolimus, and 42 were treated with MMF, one was excluded. While both treatments were generally well-tolerated, those on tacrolimus reported a higher incidence of adverse effects, including infections and gastrointestinal problems, compared to the MMF group [3]. The study found that both medications were effective in managing disease activity, with MMF showing a marginally better safety profile. Over a 12-month follow-up, both tacrolimus and MMF were comparable in terms of improving kidney function and reducing proteinuria [3]. The p-value for the comparison of disease activity between the two treatments was 0.403, suggesting no significant difference in their efficacy [3].

Tacrolimus is an immunosuppressant that exerts its effects by binding to FKBP12 (FK506-binding protein 12) within cells, forming a complex that inhibits calcineurin [3]. This inhibition prevents calcineurin from activating the nuclear factor of activated T-cells (NFAT), a transcription factor essential for the expression of genes involved in T-cell activation and proliferation [3]. Consequently, this leads to reduced production of crucial cytokines, such as interleukin-2 (IL-2), which are vital for T-cell growth and function. By targeting these pathways, tacrolimus effectively diminishes T-cell activation and proliferation, helping to manage autoimmune conditions like lupus nephritis [3].

Mycophenolate Mofetil vs Cyclophosphamide

Cyclophosphamide is an alkylating agent that works by interfering with DNA replication. It forms covalent bonds with DNA, leading to cross-linking of DNA strands and preventing their separation [19]. This process inhibits DNA synthesis and cell division, particularly affecting rapidly dividing cells such as those in the immune system [19]. By disrupting the replication of these cells, cyclophosphamide reduces the proliferation of immune cells involved in the autoimmune process, thereby helping to control inflammation and disease activity in conditions like lupus nephritis [19].

The study by Sedhain et al. (2018) is a randomized controlled trial that compared low-dose MMF with cyclophosphamide for induction therapy in lupus nephritis among a Nepalese population. The trial involved 49 patients, with 21 receiving low-dose MMF and 21 treated with cyclophosphamide, seven could not participate in the study [19]. Both treatments were associated with adverse effects; however, cyclophosphamide was linked to more frequent and severe side effects, including gastrointestinal issues and infections, compared to MMF [19]. The results indicated that MMF was as effective as cyclophosphamide in reducing disease activity and improving renal outcomes, with a p-value of 0.57 at the end of six months, showing no significant difference between the two treatments in terms of efficacy [19]. The follow-up period for the study was 12 months, during which both groups demonstrated similar improvements in renal function, and disease markers [19].

In the comparison of studies on immunosuppressive treatments for proliferative lupus nephritis, mycophenolate mofetil, and cyclophosphamide were found to be similarly effective in inducing remission, though mycophenolate mofetil often demonstrated a better safety profile with fewer adverse events.

Sirukumab

Sirukumab is a monoclonal antibody that specifically targets and inhibits interleukin-6 (IL-6), a cytokine involved in the inflammatory response. By binding to IL-6, sirukumab prevents it from interacting with its receptor, thereby disrupting IL-6 signaling pathways [20]. This inhibition reduces the production of pro-inflammatory cytokines and immune cells, helping to modulate the autoimmune activity and inflammation associated with lupus nephritis [20].

The study by Rovin et al. (2016) was a multicenter, randomized, double-blind, placebo-controlled trial evaluating the effectiveness and safety of sirukumab (CNTO 136) in treating active lupus nephritis [20]. The research included 294 participants, with a portion receiving sirukumab and the remainder receiving a placebo [20]. The sirukumab group experienced a higher incidence of adverse effects, such as infections and infusion reactions, compared to the placebo group. Although sirukumab demonstrated some ability to reduce disease activity, its efficacy did not significantly exceed that of conventional treatments like tacrolimus or MMF [20]. The study followed patients for 24 weeks indicating that the difference in efficacy between sirukumab and placebo was not statistically significant [20].

Ocrelizumab

Ocrelizumab is a monoclonal antibody that targets and depletes CD20-positive B cells. By binding to CD20, ocrelizumab induces the destruction of these B cells through mechanisms such as ADCC and CDC [21]. This reduction in CD20-positive B cells helps to decrease the production of autoantibodies and modulate the immune response, thereby mitigating the autoimmune activity and inflammation associated with conditions like lupus nephritis [21].

The research conducted by the author in 2013 was a randomized, double-blind, phase III trial aimed at evaluating both the effectiveness and safety of ocrelizumab in individuals with active proliferative lupus nephritis [21]. The trial included 223 patients, with a portion receiving ocrelizumab at doses of 400mg, and 1000mg, and a placebo followed till 48 weeks. Ocrelizumab was associated with adverse events such as infusion reactions and infections, which were more frequent compared to the placebo group [21]. The study did not provide a direct comparison between ocrelizumab and standard treatments like tacrolimus or mycophenolate mofetil. Results indicated that ocrelizumab showed some improvement in disease activity, but did not significantly outperform standard therapies [21]. The follow-up period was 24 weeks, and the p-value for the primary outcome measure of renal response was 0.065, there is a significant difference numerically, but not superior to the placebo. Additionally, it has a higher number of infection rate [21].

Atacicept

Atacicept is a recombinant fusion protein that inhibits BLyS and a proliferation-inducing ligand (APRIL) [22]. By blocking these cytokines, atacicept reduces the survival and proliferation of autoreactive B cells, which helps to decrease autoantibody production and modulate the immune response in lupus nephritis [22].

The research conducted by the author in 2012 was a randomized, controlled clinical trial designed to assess the efficacy and safety of atacicept in combination with MMF and corticosteroids for treating lupus nephritis [22]. The trial enrolled six patients. The study faced premature termination due to adverse events, including infections and infusion-related reactions, which were more common in the atacicept group [22]. Although the study aimed to compare atacicept with standard treatments like tacrolimus or MMF, it did not provide a direct comparison with these drugs. Results suggested that while atacicept had potential, the overall efficacy did not significantly surpass that of standard treatments [22]. The follow-up period was decided to be of 52 weeks, and the p-value for the primary outcome measure was not provided due to the early termination of the trial [22].

Limitations of the study

The main limitation of research is that the inherent difficulties of the complex disease mean that most studies have relatively small sample sizes and tend to raise concerns that may not be generalizable to the overall treatment outcomes for the population. Many studies are limited by the inclusion and exclusion criteria, which thus far cannot allow for the results to generalize as outcomes cannot be generalized to patients who do not satisfy that criteria. Follow-up periods are often too short to make it adequate to decide the long-term effects of therapies. Patients suffering from lupus nephritis can be geographically and demographically diverse, which can impact the generalizability of the findings, as certain findings from particular populations or regions cannot be universally applied to the remainder of patients with this condition.

Future strategies

Future studies on lupus nephritis should therefore enroll more diversified patients in the clinical trials because efficacy may be highly variable among the demographics. Such promising short-term benefits in many of the treatments require long-term safety and efficacy data best achieved with extended follow-up studies. Standardised treatment protocols will allow tailoring therapies according to individual responses. But ultimately, inclusion of cost-effectiveness analyses shall ensure that the effective treatments reach wider numbers. Collecting real-world evidence will further enhance our understanding of how effective therapy can be, and the study of early intervention timing and mechanisms of resistance can guide the development of new and improved treatments.

## Conclusions

Recent research highlights voclosporin and belimumab as leading treatments for lupus nephritis, given their robust efficacy profiles. Voclosporin is highly effective in achieving remission and lowering proteinuria while maintaining a favorable safety profile. As a newer therapeutic option, it demonstrates significant potential for managing active lupus nephritis. Belimumab also shows promising outcomes, especially when combined with other therapies, and has a commendable safety record. Its effectiveness is notably confirmed in the East Asian population, showcasing its broad applicability.

In contrast, while rituximab and obinutuzumab are effective treatments, their complex dosing schedules and potential safety issues make them less straightforward compared to newer therapies. Tacrolimus and MMF remain important, established treatments with proven efficacy. Among these options, voclosporin is frequently highlighted as the most effective based on the latest evidence besides MMF. Its high efficacy and well-tolerated nature position it as a preferred choice for lupus nephritis treatment. Nonetheless, the choice of therapy should be individualized, considering factors such as the severity of the disease, previous treatment responses, and unique patient characteristics. Ongoing research is vital to optimize treatment protocols and enhance patient outcomes in lupus nephritis.
